# Coronary Artery Fistula in an Elderly Female: A Case Report

**DOI:** 10.7759/cureus.60753

**Published:** 2024-05-21

**Authors:** Duaa Alhazmi, Rana Saklou

**Affiliations:** 1 Department of Diagnostic Radiology, King Fahad General Hospital, Jeddah, SAU

**Keywords:** coronary artery angiography, fistula, echocardiography, ct angiography, coronary artery

## Abstract

A coronary artery fistula (CAF) is an abnormal, direct connection between the coronary arteries and cardiac chambers, systemic circulation, or pulmonary circulation without an intervening capillary network. We report a case of a CAF observed in an elderly female. Congenital CAFs are indeed relatively rare, with an incidence of 0.002% to 0.3% in the general population. Over the past few decades, coronary angiography and multi-detector computed tomography have become more widely available, leading to an increased detection of asymptomatic patients with CAFs. By accurately characterizing the CAF's anatomy and understanding the risk factors for complications, clinicians can make more informed decisions about the most appropriate treatment strategy.

## Introduction

Coronary artery fistulas (CAFs) are coronary anomalies incidentally recognized in asymptomatic patients. A CAF is an abnormal, direct connection between the coronary arteries and cardiac chambers, systemic circulation, or pulmonary circulation without an intervening capillary network [1]. The most common origin of CAFs is the right coronary artery (RCA), representing 50% to 55% of cases, followed by the left anterior descending artery (LAD), accounting for 35% to 40% of cases. The left circumflex artery (LCX) is the origin of CAFs in 5% to 20% of cases [2]. The pulmonary trunk is the most common drainage site of CAFs (89%), followed by the right ventricle (41%), the right atrium (26%), the left atrium and left ventricle (3%-5%), and the coronary sinus (7%) [3].

While most patients with CAFs are asymptomatic, some patients may experience symptoms including cardiac dysfunction, right ventricular enlargement, endocarditis, arrhythmias, dyspnea, orthopnea, and chest pain [4]. From a pathophysiological perspective, a dilated and tortuous CAF could alter the normal pathway of myocardial blood flow, leading to decreased flow and, in certain instances, ischemia in downstream myocardial territories. Eventually, the pressure gradient between the coronary artery and its drainage site can cause a compensatory mechanism leading to coronary enlargement or tortuosity. This can lead to several potential complications, including aneurysm formation, atherosclerotic deposition, and, in rare cases, rupture of the fistula. Given the potential for serious complications, it is important for clinicians to be aware of these risks when managing patients with CAFs [5, 6].

In addition to the potential complications mentioned above, a CAF can also cause left-to-right shunting, leading to dilation of both the right and left ventricles. The severity of this shunting and the progressive dilation of the ventricles depends on the anatomical characteristics of the CAF, such as size, location, flow rate, and duration.

Over the years, technological advances in cardiac magnetic resonance (CMR) imaging and cardiac computed tomography (CCT) have allowed for a more accurate and comprehensive assessment of CAFs. CMR and CCT can provide detailed information on the anatomy and function of the coronary arteries, myocardial perfusion, and tissue characteristics, and can be particularly useful in asymptomatic patients who require regular and rigorous follow-up. CMR also has the advantage of being a non-invasive imaging modality that does not involve exposure to ionizing radiation.

T2-weighted CMR sequences with and without fat suppression can be utilized to evaluate inflammation and swelling, while steady-state free precession (SSFP) sequences are commonly used to visualize vascular structures and assess cardiac kinetics and function. Delayed enhancement sequences obtained 8-10 minutes post-IV administration of a gadolinium contrast agent are used to evaluate tissue characteristics. Phase-contrast sequences, on the other hand, can be used to assess blood flow, which is particularly useful in patients with suspected shunts [7].

Coronary magnetic resonance angiography (MRA) without contrast enhancement is possible using 3D balanced respiratory navigated sequences with fat suppression and T2 pulse preparation. This technique has several advantages, including the ability to visualize the coronary arteries without the need for contrast agents, a high signal-to-noise ratio, and good spatial resolution. However, it is also time-consuming and limited in its ability to visualize the distal coronary arteries. Nevertheless, advances in this field hold promise for future clinical applications [8].

## Case presentation

The case is of a 62-year-old female patient with a known history of hypertension, dyslipidemia, and hypothyroidism, who presented with atypical chest pain. She was admitted to the cardiology department as a case of stress-induced myocardial infarction with hypertensive heart disease. Lab results revealed normal cardiac enzyme levels and a normal ECG, while a stress ECG was terminated at stage II after 5 minutes due to chest pain and showed horizontal ST depression in the inferior leads and V5-V6. The test was considered positive for stress-induced MI.

**Figure 1:**
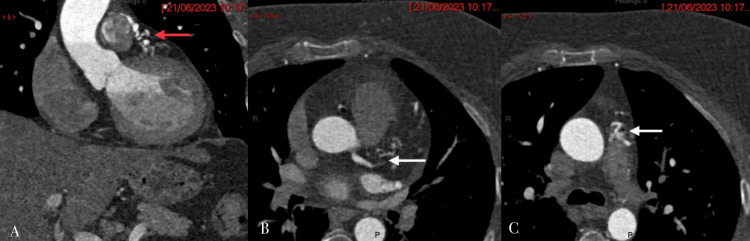
(A) Coronal and (B, C) axial CT images show multiple dilated, tortuous fistulous tracts (red arrow in A) arising from the left anterior descending artery (arrow in B) and the right conal artery, draining into the main pulmonary artery (arrow in C).

**Figure 2:**
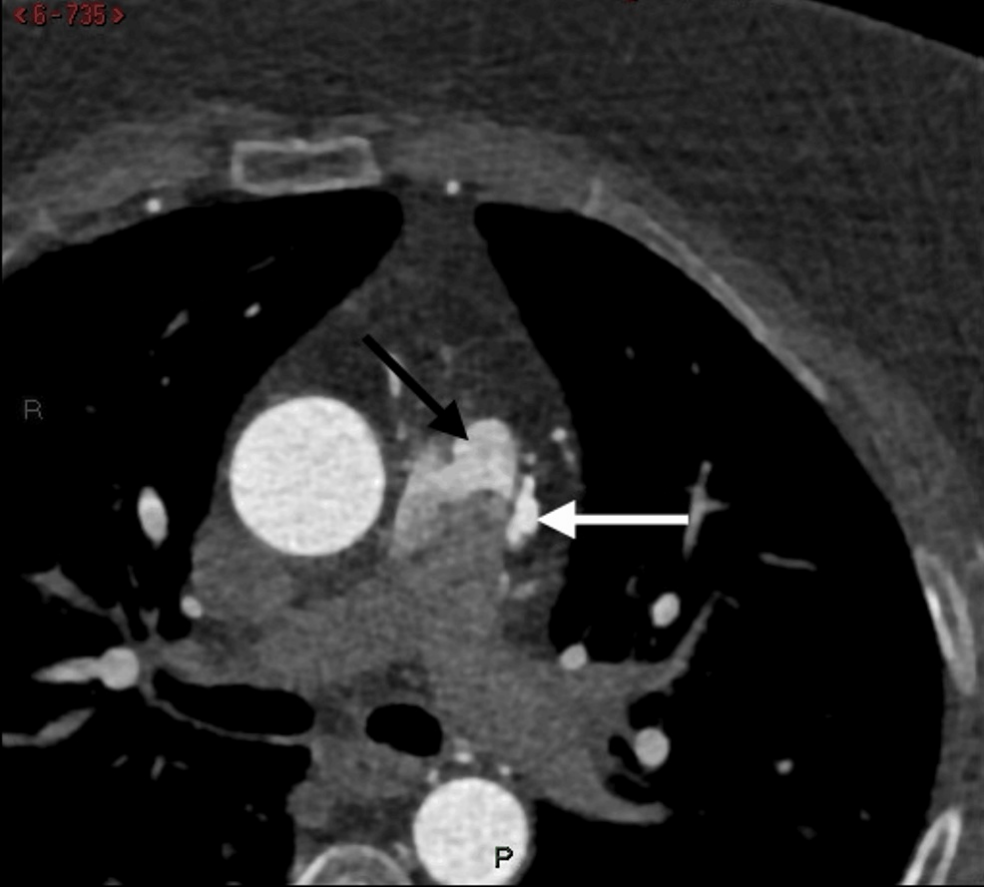
An axial CT image shows a typical pierced sign and the smoke sign, resulting from a jet of contrast flushing into the main pulmonary artery (black arrow) and small focal aneurysm formation (white arrow).

**Figure 3:**
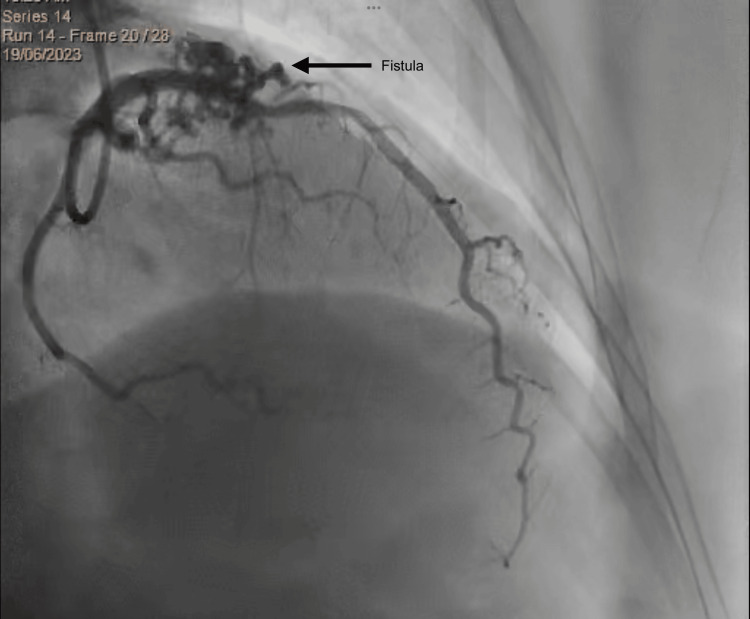
Coronary angiogram showing fistula arising from proximal left anterior descending artery to the main pulmonary artery.

Echocardiography showed normal left ventricle dimensions, mild left ventricular hypertrophy with good systolic function (EF 57%), and no regional wall motion abnormalities (RWMA) at rest. However, diastolic dysfunction grade I was noted, along with trivial mitral regurgitation and tricuspid regurgitation. Coronary CT angiography revealed normal origin, course, and termination of the coronary arteries with right dominance and a calcium score of 0. The coronary arteries' lumen was unremarkable without plaques or stenoses. However, there were worm-like tortuous dilated fistula vessels originating from the left anterior descending artery and the right conal artery, which had a separate origin from the aorta, accompanied by focal aneurysm formation. Additionally, a fistula vessel was draining into the left lateral wall of the main pulmonary artery, with a jet of contrast from the supplying artery to the main pulmonary artery forming a typical pierced sign and the smoke sign (Figures 1-2). Left ventricular angiography showed a right dominant coronary system, normal left main, and normal LAD artery. However, there were multiple small fistulae branches from the proximal LAD draining mostly to the pulmonary artery, and a small fistula from the conus branch to the pulmonary artery (Figure 3).

Accordingly, no interventional therapy was performed for the patient, and she was shifted to non-invasive management with the use of antiplatelet therapy and close monitoring.

## Discussion

Congenital CAFs are indeed relatively rare, with an incidence of 0.002% to 0.3% in the general population. Over the past few decades, coronary angiography and multidetector computed tomography have become more widely available, leading to an increased detection of asymptomatic patients with CAFs.

This case report provides a comprehensive outlook on a female patient with coronary artery fistulas. Invasive coronary angiography has been the gold standard imaging technique for diagnosing CAFs, but its limitations include the inability to accurately visualize large and/or high-flow CAFs and the surrounding structures. This can lead to an underestimation of the course of the fistula or difficulty visualizing it. However, in this case, coronary CT angiography was adopted and revealed a normal origin, course, and termination.

While intervention with either surgical or transcatheter closure is often technically feasible, there has been a shift towards conservative management in asymptomatic patients due to the potential risks of intervention, such as post-closure sequelae like residual leaks, thrombosis, and coronary stenosis [4]. Therefore, there has been a shift from intervention towards watchful waiting in asymptomatic patients.

Spontaneous closure of a CAF is uncommon, occurring in only 1% to 2% of cases, so regular follow-up is recommended [1]. For asymptomatic patients with small CAFs, the recommended management is typically non-invasive, with the use of antiplatelet therapy and antibiotics, and close monitoring for any complications that may arise [9].

The American College of Cardiology (ACC) and American Heart Association (AHA) guidelines recommend interventional management for large CAFs regardless of the presence of symptoms. For small-to-moderate CAFs, interventional management is recommended if symptoms such as myocardial ischemia, arrhythmia, ventricular dysfunction, or endarteritis are present. Surgical ligation and percutaneous transcatheter closure are the two main treatment options for patients who require intervention [10].

The importance of collaboration among various specialties cannot be overstated when it comes to selecting the most appropriate strategy for managing complex patients with CAFs. Blood flow characteristics such as rate, velocity, and turbulence are known to change after fistula closure, which can increase the risk of intracoronary thrombosis in the aneurysmal segment. As such, close follow-up of patients who undergo fistula closure is essential to detect any complications such as coronary thrombosis and myocardial ischemia.

## Conclusions

CAF could alter the normal course of myocardial blood flow, leading to decreased flow and, in certain instances, ischemia in downstream myocardial territories. By accurately characterizing the CAF's anatomy and understanding the risk factors for complications, clinicians can make more informed decisions about the most appropriate treatment strategy.
